# Non-Necrotizing Bullous Cellulitis and Bacteremia: A Rare Presentation of the Shewanella Algae Infection

**DOI:** 10.7759/cureus.12776

**Published:** 2021-01-18

**Authors:** Niranjan Ojha, Kevin Walsh, Matthew J Hess

**Affiliations:** 1 Internal Medicine, Upstate University Hospital, Syracuse, USA; 2 Hospital Medicine, Upstate University Hospital, Syracuse, USA

**Keywords:** nonnecrotizing bullous cellulitis, shewanella alga, bacteremia

## Abstract

A 58-year-old male with severe psoriasis on Risankizumab presented with painful, left leg swelling with erythema and blisters concerning for necrotizing fasciitis. Intraoperative findings showed non-necrotizing bullous cellulitis. The blood and tissue cultures grew *Shewanella *algae. A handful case of non-necrotizing bullous cellulitis has been reported but this is the first documented case of non-necrotizing bullous cellulitis and bacteremia in PubMed.

## Introduction

*Shewanella* species are a group of bacteria frequently isolated from salt and fresh water as well as food and human sewage. This is a nonlactose fermenting Gram-negative marine bacillus that produces hydrogen sulfide as one of its most prominent features [1-2]. *Shewanella* spp., although still relatively rare, are becoming an increasingly recognized cause of human infections including cellulitis, bacteremia, and necrotizing fasciitis with a predilection for certain sub-population such as immunocompromised individuals, individuals with chronic skin breakdown. No prior case of bullous cellulitis caused by *Shewanella* was previously reported in the literature. Here, we present possibly the first documented case of bullous cellulitis with bacteremia secondary to *Shewanella* spp. infection [3-6].

## Case presentation

**Figure 1:**
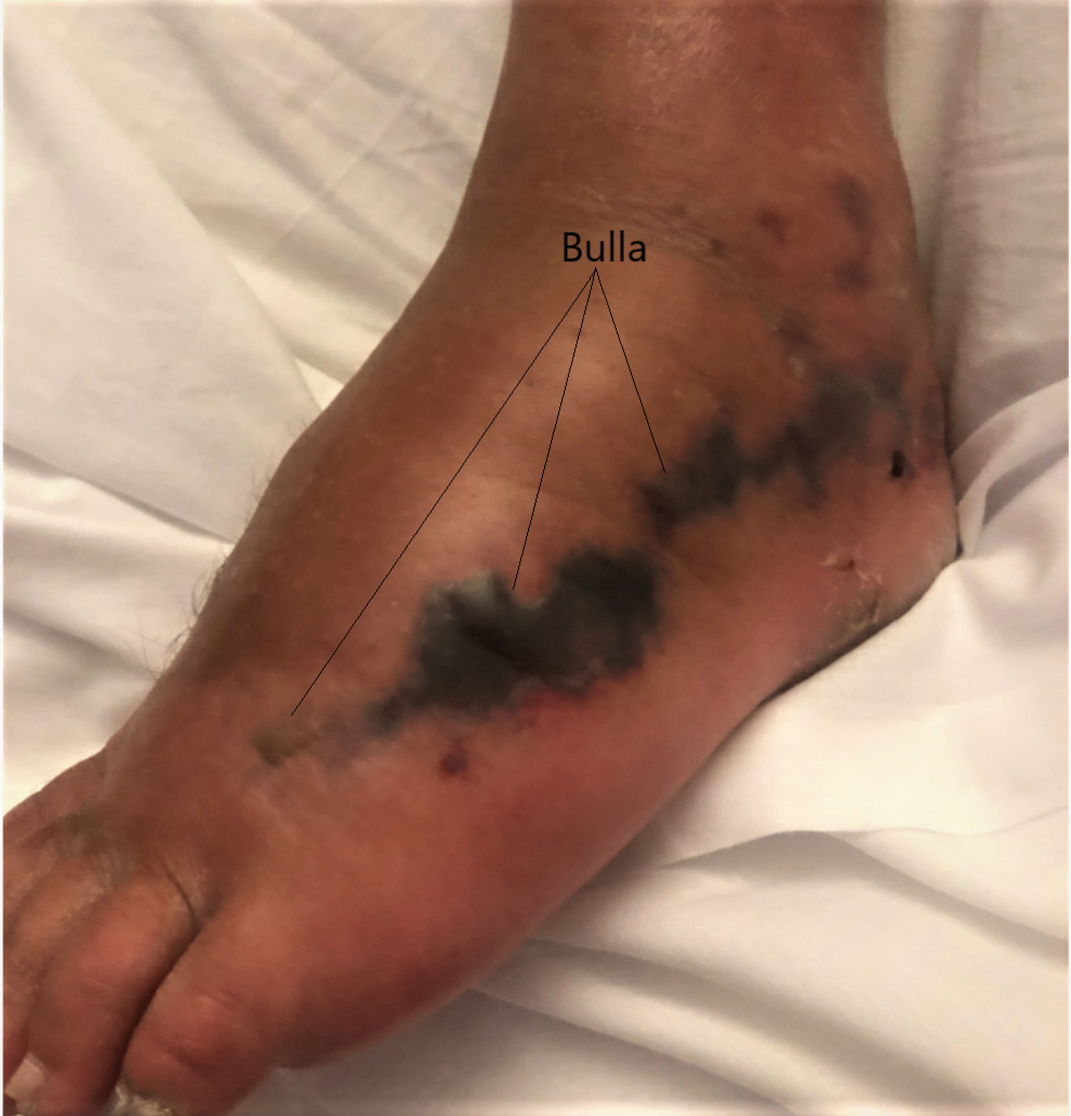
Bulla with black discoloration of the wound, present on admission.

A 58-year-old male with a past medical history of atopic dermatitis and severe psoriasis on Risankizumab (Skyrizi) presented with a chief complaint of progressive left leg swelling for a week associated with erythema and pain. The patient had a chronic history of skin breakdown on his bilateral legs due to psoriasis. He was vacationing in Belize and was walking barefoot on the ocean beaches. Initially, he noticed a burning sensation and increased redness of the left lower limb when he was still in Belize. The swelling, pain, and erythema progressed. The blisters developed a week after initial symptoms (see Figure 1) and two days before the arrival in the emergency room. He did not report any fever and chills.

 On physical examination, he was afebrile with a temperature of 36.8 degree celsius. He was hypotensive with a blood pressure of 88/61 mmHg and had tachycardia with a pulse rate of 114 beats per minute. He had bilateral lower limb erythema, with swollen and tender left leg, and blisters on the lateral aspect of the left leg. He had mild leukocytosis with a white blood cell count of 12,300 cells/microliters with an absolute neutrophil of 8.17(*10^3 cells/microliter). His blood culture showed many Gram-negative rods. The point of care lactate was 2.8 and the sepsis protocol was initiated with IV fluid resuscitation and broad-spectrum antibiotics. The ultrasound Doppler of the bilateral lower limbs ruled out deep vein thrombosis (DVT). He was admitted to the medical ICU for further evaluation and management. The Infectious Disease team was consulted for antibiotics management.

**Figure 2:**
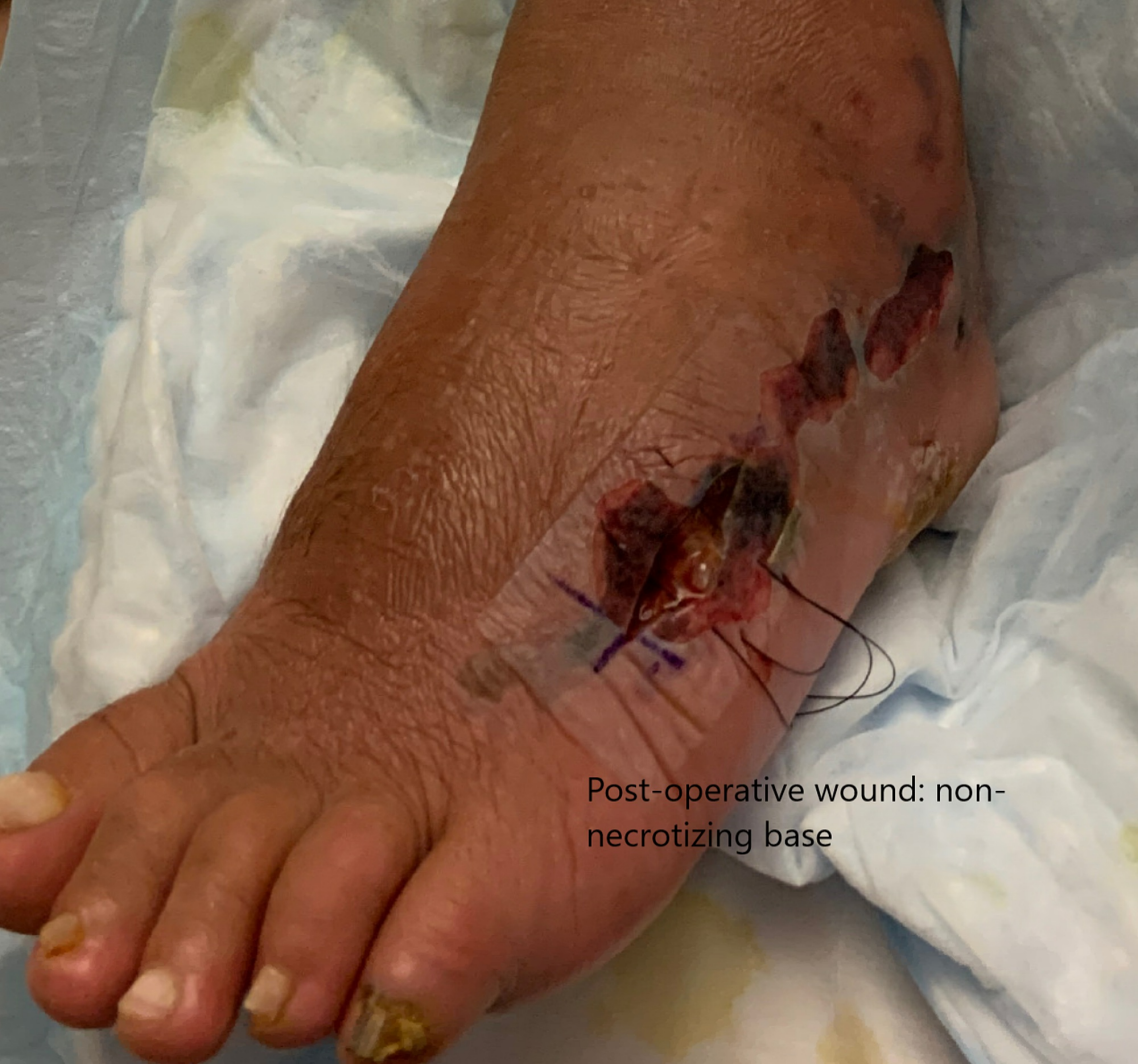
Post-operative wound showing non-necrotizing base.

The General Surgery was consulted for the clinical concern of necrotizing infection. The CT scan showed no obvious abscess or gas in the subcutaneous tissue. The patient complained of the disproportionate pain in comparison to the erythema and then developed hemorrhagic bullae in the interim. His clinical condition did not improve, and his pain was difficult to control. He underwent surgical intervention for the clinical suspicion of necrotizing fasciitis. The intraoperative findings were consistent with non-necrotizing bullous cellulitis (see Figure 2).

The blood culture differentiated into *Shewanella* algae. The wound culture and intraoperative tissue culture also grew *Shewanella* algae. The patient was initially started on ceftazidime and ciprofloxacin. The micro-organism was pan-sensitive (see Table 1), including ciprofloxacin, and the subsequent blood culture from day 2 did not demonstrate any bacterial growth. The patient was discharged on oral ciprofloxacin 750 mg twice a day for 14 days. The patient was seen in the outpatient Infectious Disease clinic after 10 days. The wound was minimally draining and the decision was made to extend antibiotics therapy by one week.

**Table 1:** Culture/sensitivities table for Shewanella algae. MIC, minimum inhibitory concentration

Antibiotics	Alternate MIC susceptibilities
Ceftazidime	0.38 Sensitive
Ciprofloxacin	0.19 Sensitive
Gentamicin	0.75 Sensitive
Piperacillin + Tazobactam	0.38 Sensitive
Tobramycin	1.00 Sensitive

Unfortunately, the patient was re-admitted for reinfection of the wound with methicillin-resistant Staphylococcus aureus (MRSA) infection. He required wound debridement and the Infectious Disease team recommended a prolonged course of antibiotics. He completed six weeks of IV vancomycin and oral ciprofloxacin following his wound debridement. The wound that was left to heal by secondary intention was healing well and >90% closed in his last Infectious Disease clinic follow up. The IV antibiotics were discontinued at that time.

## Discussion

The Gram-negative *Shewanella* spp. are widespread in the marine environment. They are mostly innocuous to human health and rarely pathogenic but known as spoilage bacteria of fish [5]. The species *putrefaciens* is the first species reported in the *Shewanella* genus. When the initial species was identified it was named *Achromobacter putrefaciens*. The species was further studied and reclassified as *Pseudomonas putrefaciens*. These organisms are reclassified into the family Vibrionaceae and got their new name *Shewanella*, named after James Shewan for his work in marine microbiology [1, 5-7].

*Shewanella putrefaciens* and *Shewanella* algae are the common species associated with human infections. Many *Shewanella* infections in the literature are attributed to *S. putrefaciens*. Nozue et al. re-identified the *S. putrefaciens* infections as *Shewanella* algae infections in his 1992 paper, so, *Shewanella* algae infections may have been less reported in the literature [7]. In the laboratory, *Shewanella* algae can produce beta hemolysis in the blood agar, can grow at 42 degree celsius, can grow in NaCl 6%, and also canreduce nitrite, whereas *S. putrifaciens* cannot [1, 7-8].

Our patient had a recent trip to a warmer place with a subtropical climate with sea beaches that put him at risk of exposure to the *Shewanella* spp. which are in abundance in the marine ecosystem.

The common infections associated with *Shewanella* spp. are infection of the ear, respiratory tract, skin and soft tissue, abdominal or biliary tract with or without bacteremia. The bacteremia is most commonly associated with skin and soft tissue infection and abdominal or biliary tract infection [1, 5]. Our patient presented with progressive left leg swelling with erythema and blisters associated with moderate-severe pain. The patient had bacteremia and was septicemic on presentation.

The portals of entries reported in the literature include leg ulcers, chronic skin breakdowns, superinfection of open fractures, and superinfection of pre-existing blisters [5]. Our patient has psoriasis and has chronic skin breakdown which likely was a portal of entry. The superinfection of pre-existing blisters has been reported in a handful of literature but our patient developed blisters after a week of initial symptoms of limb swelling, erythema, and pain. We searched Google Scholar and PubMed library for existing literature reporting the development of blisters following *Shewanella* infection but we could not find any such literature.

In one literature review of *Shewanella* spp. infections, 11% were immunocompromised due to various underlying reasons, including cancer with chemotherapy, steroid use, splenectomy, neutropenia, HIV infection, and immunosuppressive drugs. There have been reports of primary bacteremia with a fulminant course with underlying liver disease and malignancy [1, 5, 9].

Our patient was receiving immunotherapy treatment with Risankizumab for psoriasis. The abscess (in the neck) and bacterial meningitis have been reported as serious infection during the phase III randomized controlled trial of the Risankizumab [10]. The immunotherapy for psoriasis could have put him at the risk of infection and/or disease progression due to immunosuppression. The Risankizumab was later discontinued by his dermatologist.

*Shewanella* algae and *S. putrefaciens* are susceptible to aminoglycosides, carbapenems, erythromycin, and quinolones [1]. Our patient was initially treated with ceftazidime and ciprofloxacin. Once culture and sensitivity showed *Shewanella* algae sensitive to ciprofloxacin, only ciprofloxacin was continued thereafter.

## Conclusions

*Shewanella* species infections have myriads of presentation and are known to cause skin and soft tissue infection with and without bacteremia. The cellulitis and necrotizing fasciitis have been reported but this would be the first reported case to our knowledge of non-necrotizing bullous cellulitis caused by *Shewanella* algae. It is one more condition, albeit rare to consider for a clinician evaluating a case of bullous cellulitis.
